# Identification and Verification of Key MiRNAs Associated with Intervertebral Disc Degeneration

**DOI:** 10.2174/1386207325666220915113438

**Published:** 2023-04-27

**Authors:** Jianwei Liu, Rong Li, Peizhen Lyv

**Affiliations:** 1 Department of Osteology, The Second Nanning People’s Hospital, The Third Affiliated Hospital of Guangxi Medical University, Nanning, 530031, Guangxi, China;; 2 Department of Reproductive Center, The Second Nanning People’s Hospital, The Third Affiliated Hospital of Guangxi Medical University, Nanning, 530031, Guangxi, China

**Keywords:** Degeneration, intervertebral disc, microRNAs, gene expression, gene regulatory networks, WGCNA

## Abstract

**Background:**

Intervertebral Disc Degeneration (IDD) is a heterogeneous spinal disease whose underlying molecular mechanism is unclear.

**Objectives:**

This study aimed to identify, profile, and analyze microRNAs (miRNAs) related to IDD.

**Methods:**

Microarray Gene Expression IDD data (GSE63492) were downloaded from Gene Expression Omnibus datasets. We employed Weighted Gene Co-Expression Network Analysis (WGCNA) to construct a miRNA co-expression network, and the miRNAs related to the IDD stage were detected. The number of differentially expressed miRNAs between normal and degenerated nucleus pulposus tissues was calculated. Twenty-three clinical specimens were used to validate the expression of miRNAs using qRT-PCR.

**Results:**

WGCNA identified 48 miRNAs significantly related to the IDD stage, and 94 miRNAs that were significantly different between normal and degenerated nucleus pulposus tissues. We selected 32 overlapping miRNAs and identified 347 corresponding target genes. The integrative analysis revealed the biological function and pathways of these targeted genes. Analysis of clinical specimens validated that hsa-miR-4534 was upregulated in IDD, whereas hsa-miR-1827 and hsa-miR-185-5p were downregulated in IDD.

**Conclusion:**

This study has identified a subset of miRNAs that are related to IDD pathogenesis and hub miRNAs that are keys to the IDD co-expression network, which may potentially be utilized as indicators for treatment.

## 1 INTRODUCTION

Intervertebral Disc Degeneration (IDD) is a common cause of lower back pain in most individuals over 50 years of age [1]. Current treatment for late-stage IDD with severe neurological symptoms is largely limited to pharmacotherapy or surgical intervention, but the effectiveness of these methods remains controversial. The ineffectiveness of these methods can partly be attributed to our limited information regarding the pathogenesis of IDD [2]. Hence, a better understanding of IDD pathogenesis will enable more effective therapies for IDD patients. In the past decades, increasing research evidence has revealed that IDD is a multifaceted spinal disease, in which aging, certain diseases, injuries, and genetic predisposition contribute to the development of IDD. To date, changes in molecular expression are also considered to be a crucial contributor to the development of IDD, and several coding genes associated with IDD have been identified [3]. In addition to the coding genes, there has recently been increasing attention to the effect of non-coding RNAs in the pathogenesis of IDD [4, 5].

MicroRNAs (miRNAs) are short non-coding RNAs that play important roles in various biological cellular processes, including cell differentiation, apoptosis, and proliferation, by regulating the expression of their target genes. Several miRNAs can regulate the biological function of intervertebral disc cells and potentially contribute to IDD development [6-8]. High-throughput technologies, including microarray, RNA-sequence and metagenome sequencing, have recently been developed and facilitated in generating several expression datasets involving coding genes, non-coding RNAs and other spectra associated with various diseases and biological processes [9-14]. To date, there are also some microarray datasets of IDD that are available. For example, Fan *et al.* provided a few potential networks among competing endogenous RNAs (ceRNAs) related to IDD and mainly emphasized the interactions among lncRNAs, miRNAs, and mRNAs [9]. However, no clinical validations were done. Therefore, in the present study, we comprehensively analyzed IDD microarray datasets to establish miRNA profiles closely related to IDD pathogenesis. In addition, this study also utilized a large number of clinical specimens to validate miRNA profiling results, which may potentially serve as therapeutic targets for IDD treatment.

## 2 MATERIALS AND METHODS

### 2.1 Microarray Data

The GSE63492 miRNA expression dataset [15] was obtained from the National Center of Biotechnology Information (NCBI) Gene Expression Omnibus (GEO) database. This dataset consists of 10 samples, including 5 degenerated nucleus pulposus tissues and 5 normal nucleus pulposus tissues. The microarray data were generated based on the GPL19449 platform [(Exiqon miRCURY LNA microRNA Array, 7^th^ generation REV - hsa, mmu, and rno (miRBase v18.0)]. We obtained degenerative nucleus pulposus samples from IDD patients. Non-degenerative specimens were collected from cadaveric donors. Samples gathered from graded I (Pfirrmann grading) [16] cadaveric donors were defined as the normal group, whereas patient samples graded IV or V belonged to the IDD group.

### 2.2 Co-Expression Network Analysis and Module-Trait Associations of miRNAs

A co-expression network of miRNAs was built by weighted gene co-expression network analysis (WGCNA) [17] using the R language and following the WGCNA protocol. Briefly, Pearson’s scores of all miRNAs for the relation matrix were calculated and transformed by raising all values to a power beta value. Using the transformed correlation matrix, we calculated a topological overlap matrix that was then converted into a dissimilarity matrix, thereby creating a hierarchical cluster tree. We identified miRNA co-expression modules in the hierarchical cluster tree with a dynamic tree cut procedure. We estimated module-trait associations using the correlation between the phenotype and module eigengene, which allowed the identification of modules that were highly correlated to the phenotype. In each expression profile, the significance of the miRNAs was determined to be the absolute correlation value between the expression profile and each trait [18]. To identify miRNAs in each module closely related to the clinical stage, we used the gene significance (GS) to estimate the correlation of miRNAs to the phenotype. GS refers to the absolute value of the correlation between a clinical trait and a specific gene/miRNA [17].

### 2.3 Differential Analysis of miRNAs and Overlapping miRNAs

The GSE63492 data were initially pretreated using the Bioconductor package in the R language prior to differential analysis. Briefly, we converted the probe-level data into expression values. Then, the expression values of all probes in every sample were reduced to a single value by calculating the average expression value. Any missing data were imputed, followed by quantile normalization of the complete data. The differential expression of miRNAs between normal and degenerated nucleus pulposus tissues was estimated using a Student’s *t*-test implemented in the LIMMA package [19]. The miRNAs with the logFC > 0.5 and p-value < 0.05 were defined as differentially expressed miRNAs (DEmiRs). The miRNAs related to the IDD stage (stage-related miRNAs) and miRNAs from the differential analysis were merged to identify miRNAs (overlapping miRNAs) that were significantly associated with the development of IDD.

### 2.4 Integrative Analysis of Predicted Target Genes Overlapping miRNAs

The target genes of the overlapping miRNAs were queried in the databases: TargetScan [20], miRDB [21], and miRTarBase [22], and genes detected in all three databases were deemed predictive target genes of every miRNA. A regulatory network of miRNA and its target gene was constructed using the combined databases and visualized through Cytoscape software version 3.5.1 [23]. Functional annotation using GO and pathway enrichment analysis with KEGG were performed and visualized using Cytoscape with ClueGo (v2.5.0) plug-ins [24]. Statistical significance was deemed at a p-value < 0.05.

### 2.5 Validation of Overlap miRNAs using Clinical Tissues

Validation experiments were performed to examine miRNA expression in intervertebral disc tissues and were approved by the ethics committee of the Third Affiliated Hospital of Guangxi Medical University. The protocol for collecting intervertebral disc tissues followed the World Medical Association Declaration of Helsinki Ethical Principles for Medical Research Involving Human Subjects [25]. In addition, written informed consent was provided by all patients. All study participants assessed from December, 2017, to April, 2018, underwent a magnetic resonance imaging (MRI) examination of the lumbar spine and were evaluated in terms of the degree of intervertebral disc degeneration following the Pfirrmann grading scheme [16]. Briefly, Grade I features a disc that is homogeneous and with bright hyperintense white signals and normal disc height. For Grade II, the disc is inhomogeneous, yet with hyperintense white signals, the annulus and nucleus are clearly differentiated, possibly with a gray horizontal band, and disc height is normal. In Grade III, the disc is inhomogeneous coupled with intermittent gray signal intensity, the nucleus and annulus are not distinct, and disc height is normal or slightly decreased. In Grade IV, the disc is inhomogeneous and with hypointense dark gray signal intensity, the nucleus and annulus are more distinct, and disc height is slightly or moderately reduced. In Grade V, the disc is inhomogeneous and shows hypointense black signal intensity, nucleus and annulus show no difference, and disc space is collapsed

**Fig. (1):**
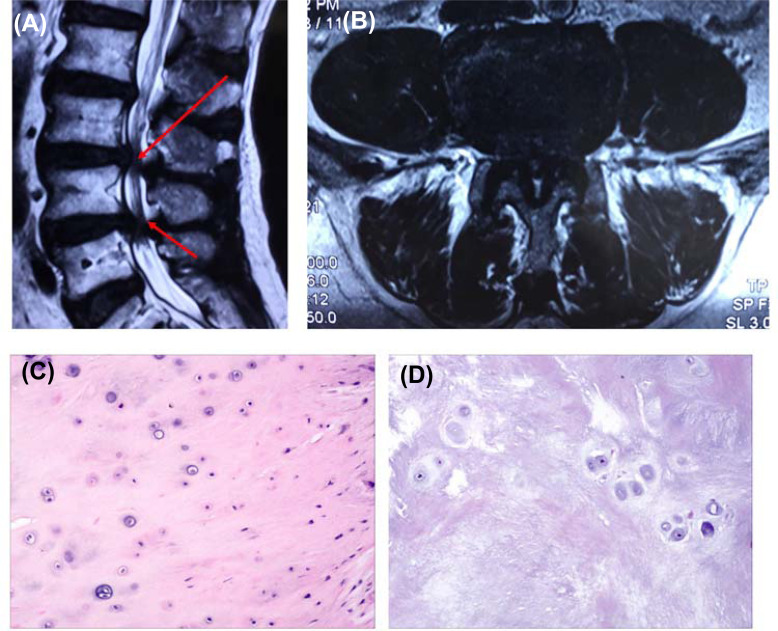
Assessment of clinical specimens. **(A)** Patient with IDD Pfirrmann grading V (sagittal T2-weighted MR image); **(B)** Patient with IDD Pfirrmann grading V (axial T2-weighted MR image of the L4-L5 disc); **(C)** Histologic features of a normal intervertebral disc; **(D)** Histologic features of an intervertebral disc with Pfirrmann grade V.

The protocol for the detection of intervertebral discs using MRI was discordant with that of a previous report [26]. This study used a 3.0-Tesla whole-body MRI (Tim Trio, Siemens Medical Solutions, Erlangen, Germany) equipped with a dedicated 8-channel spine coil (3 T Spine Matrix Coil, Siemens). Every segment was scanned using three slices, and the scanning direction and transverse plane of the intervertebral discs were parallel. All images were assessed by a well-trained radiologist and a highly experienced orthopedic spine surgeon and in consensus. There were 18 IDD tissues and 5 control intervertebral disc tissues. All intervertebral disc tissues were freshly obtained during the operation and reviewed by a pathologist (Fig. **1**).

### 2.6 RNA Extraction and Quantitative Reverse Transcription PCR (qRT-PCR) Analysis

Total RNA was extracted from fresh intervertebral disc tissues (18 IDD tissues and 5 control intervertebral disc tissues) with TRIzol regent (Aidlab, Beijing, China) following the manufacturer's instruction. RNA purity and concentration were determined by an ultraviolet spectrophotometer (Shunyu, Shanghai, China). A sequence of each hsa-miR-1827, hsa-miR-4534, and hsa-miR-185-5p was retrieved from miRbase database. The primers of three miRNAs are listed in (Table **1**). qRT-PCR was performed to quantify miRNA expression on a 7500 real-time PCR instrument (Applied Biosystems, Foster City, CA, USA) using a final volume of 20 μL. The reaction conditions included 1 cycle at 95°C for 5 minutes; 15 cycles at 95°C for 25 seconds, 64°C for 20 seconds, 72°C for 20 seconds; and a final 31 cycles at 93°C for 25 seconds, 64°C for 20 seconds, 72°C for 20 seconds. Relative miRNA expression levels were analyzed using the ^∆∆^CT method with data normalization to U6 expression.

### 2.7 Statistical Analysis

All data were presented by mean±SD. Numeric data were analyzed using the Students’ t-test. Non-numeric data were analyzed using the chi-square test. Statistical differences were taken when the p-value was under 0.05

## 3 RESULTS

### 3.1 Co-Expression Network Analysis and Module-Trait Associations using miRNAs

**Fig. (2):**
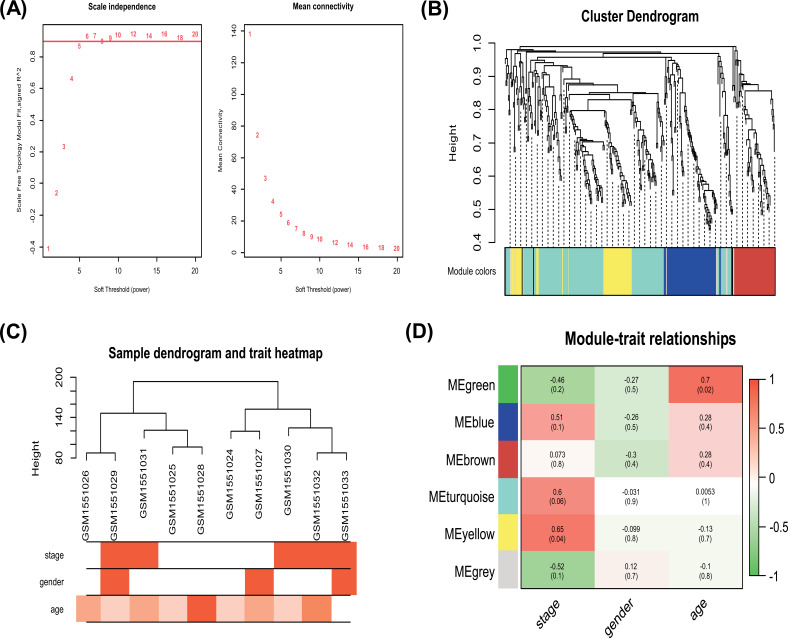
WGCNA results of miRNAs. **(A)** Selection of soft-thresholding power for WGCNA analysis; **(B)** Sample dendrogram and trait heat map; **(C)** Clustering dendrograms of the miRNAs; **(D)** Module-trait correlations of miRNAs and clinical trait; in every cell, top values are correlation coefficients of module eigengene to the traits, whereas bottom values are the corresponding p-values. Correlation coefficients closer to zero have weaker correlations, while values closer to positive or negative ones have stronger correlations. P-values less than 0.05 means significant correlation.

Using a WGCNA approach, we constructed a miRNA co-expression network for all miRNAs in the GSE63492 dataset. We selected seven miRNAs as soft-thresholding powers to construct a miRNA co-expression network (Fig. **2A**). Four significant modules were identified using the WGCNA approach; the colors of these modules were brown, green, turquoise, and yellow (Fig. **2B**). By module-trait analysis of WGCNA, we analyzed the clinical features of IDD patients (*i.e*., age, sex, and stage) with the miRNAs (Fig. **2C**). Six modules were identified; only the yellow module was associated with the IDD stage (*p* = 0.004). Table **S1** shows the miRNAs that are significantly associated with the IDD stage. However, no significant correlation was observed between sex and all the modules (Fig. **2D**).

### 3.2 Differentially Expressed miRNAs in IDD Tissues

**Fig. (3):**
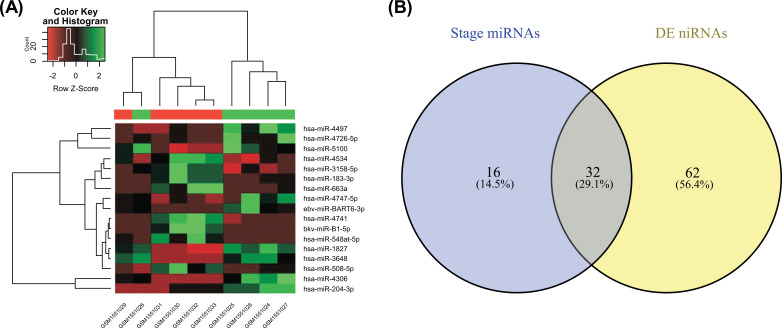
**(A)** Heat map showing the hierarchical clustering of differentially expressed miRNAs identified between normal and degenerated nucleus pulposus tissues. **(B)** The overlapping miRNAs between stage-related miRNAs and differentially expressed miRNAs.

Using selected criteria (logFC >0.5 and *P* value <0.05), we identified 94 miRNAs that were differentially expressed compared to normal and degenerated nucleus pulposus tissues, of which 38 miRNAs were upregulated and 56 were downregulated (Table **S2**). The heat map of the top 20 differentially expressed miRNAs is presented in (Fig. **3A**). Overlapping miRNAs were obtained by merging the stage-related miRNAs from WGCNA and differentially miRNAs, and 32 overlapped miRNAs were finally identified (Fig. **3B**).

### 3.3 Integration Analysis of Overlap miRNAs Target Genes

**Fig. (4):**
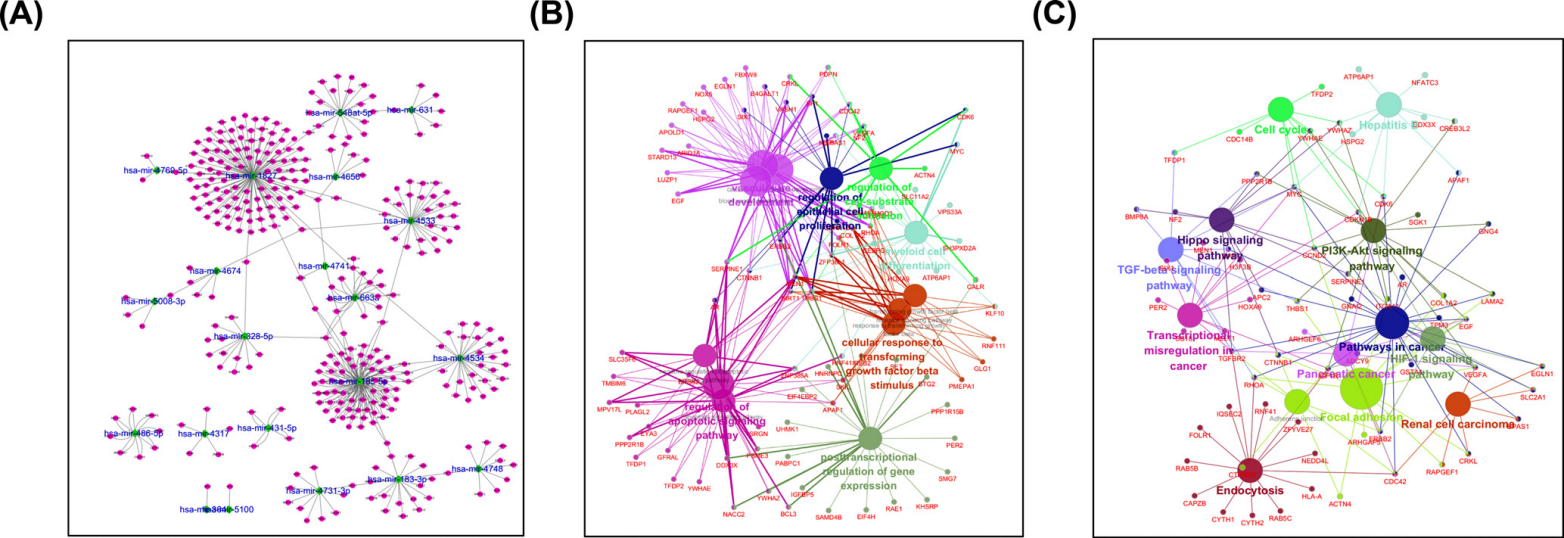
Integrative analysis of target predictions. **(A)** Correlation between miRNAs and target genes. **(B)** Functional annotation of the target genes; **(C)** KEGG pathway analysis of identified target genes.

By searching the databases TargetScan, miRanda, and MirTarget, we identified 347 target genes of the overlapping miRNAs, and the miRNA–mRNA interaction is depicted in **(**Fig. **4A**). Further analysis using ClueGo revealed that the target genes were of the following biological functions: posttranscriptional regulation of gene expression (GO:0010608), myeloid cell differentiation (GO:0030099), and regulation of cell-substrate adhesion (GO:0010810) (Fig. **4B**). The Kyoto Encyclopedia of Genes and Genomes (KEGG) pathways are as follows: HIF-1 signaling pathway (KEGG:04066), cell cycle (KEGG:04110), and hepatitis B (KEGG:05161) (Fig. **4C**).

### 3.4 Validation of Overlapping miRNAs using Clinical Specimens

**Fig. (5):**
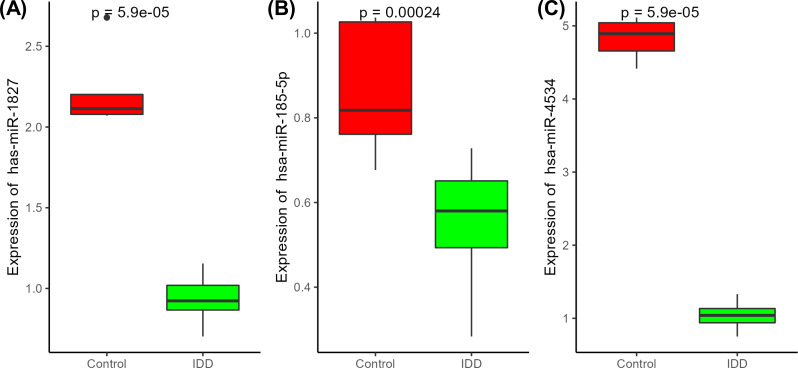
Comparison of miRNAs expression in control tissues and IDD tissues. **(A)** hsa-miR-1827; **(B)** hsa-miR-4534, **(C)** hsa-miR-185-5p. IDD: Intervertebral disc degeneration.

We selected three miRNAs (hsa-miR-1827, hsa-miR-4534, and hsa-miR-185-5p) that had the highest number of the targeted genes and were validated by qRT-PCR assay with clinical specimens. There were 23 intervertebral disc tissues, including 18 IDD tissues and 5 control tissues, with no significant differences between IDD and controls regarding patient age and sex (Table **2**). qRT-PCR analysis revealed that the relative expression of hsa-miR-4534 markedly increased in IDD compared to controls, while the expression levels of hsa-miR-1827 and hsa-miR-185-5p decreased in IDD compared to controls (Fig. **5**; *p* < 0.01). These findings coincided with the results of miRNA profiling.

## 4 DISCUSSION

To date, no effective treatment approaches are available for IDD patients with severe neurological symptoms. Hence, finding alternative approaches, such as genetic therapies, to slow or reverse the process of disc degeneration is imperative. A previous study confirmed that the aberrant expression of genes is associated with IDD pathogenesis [27], while miRNAs regulate the expression of the target genes. Thus, miRNAs may potentially be utilized as suitable therapeutic targets in IDD. The biological function of miRNAs has been well-defined in some diseases; however, the contribution of miRNAs to IDD remains unclear [28]. In this study, we first utilized the WGCNA method to identify the group of miRNAs that are related to the development of IDD, and then we conducted a differential expression analysis to identify another group of miRNAs that are significantly differentially expressed between normal and degenerated nucleus pulposus tissues. The overlapping miRNAs of the aforementioned two groups were then analyzed using integrative analysis, which provided information on their functions. Finally, we validated the expression profile of the three miRNAs, namely, hsa-miR-1827, hsa-miR-185-5p, and hsa-miR-4534, using clinical specimens, which further confirmed the role of these miRNAs in the pathogenesis of IDD.

WGCNA assesses similarities in expression profiles of samples and identifies highly co-expressed genes that are proximally connected in the co-expression network [29, 30]. In addition, this approach is able to divide the hub genes or miRNAs into several modules according to their relation, and the association of each module with a sample trait can be detected, thereby facilitating the identification of miRNAs that are closely related to a specific clinical trait. Previous studies have used this method to identify miRNAs that are related to the development of diseases, such as gastric cancer [31] and pancreatic cancer [32]. Here, WGCNA was employed for analysis of the co-expression network of miRNAs in normal and degenerated nucleus pulposus tissues, resulting in the identification of four significant modules that are highly co-expressed and possibly possess similar biological functions. We then examined the correlations between the miRNAs and clinical traits and identified several miRNAs associated with the IDD stage.

Analysis of differentially expressed miRNAs revealed that most of the miRNAs in degenerated nucleus pulposus tissues are upregulated compared to normal nucleus pulposus tissues. To identify key miRNAs that are related to the IDD, overlapping miRNAs between the two miRNA sets were analyzed, followed by the prediction of their target genes and functional annotation. Gene ontology (GO) analysis revealed the biological function of the miRNA target genes, and KEGG analysis uncovered cell cycle-related pathways, indicating that these miRNAs may affect the process of IDD by regulating the target genes. Aberrant cycles can lead to apoptosis of nucleus pulposus cells [33]; hence, we inferred that the overlapping miRNAs regulate the cell cycle of nucleus pulposus cells that, in turn, influence IDD development. However, this hypothesis requires confirmation using *in vivo* and *in vitro* experiments.

The present study found that hsa-miR-1827, hsa-miR-4534, and hsa-miR-185-5p are significantly associated with IDD development. However, recent studies have only mainly explored their role in cancers. For example, hsa-miR-1827 has been reported to be downregulated in highly invasive lung cancer sub-cell lines, and the overexpression of miR-1827 inhibits lung cancer cell migration via targeting RBX1 and CRKL [34]. In addition, hsa-miR-1827 overexpression also results in cell cycle progression and decreases the apoptotic rate of colorectal carcinoma cells [35]. The function of hsa-miR-185-5p has also been investigated in various diseases, and studies have shown that the expression of miR-185 in prostate cancer tumor tissues and cell lines is significantly downregulated, whereas it upregulates BCL2 and BCL2L1 genes [36]. miR-185-5p is also capable of modulating cisplatin chemosensitivity of human non-small cell lung cancer by targeting ABCC1 [37]. Similar to cancer progression, IDD development compromises various supplements, such as physiological disorders, nutrient transportation impediments, extracellular matrix abnormal synthesis, and metabolic abnormalities [38], in which miR-1827, miR-4534, and miR-185-5p play major roles. The role of these miRNAs thus requires further investigation.

The present study performed a comprehensive analysis of the miRNA profiles in IDD using microarray data, thereby improving our understanding of their involvement in IDD pathogenesis by regulating target genes. However, some clear limitations need to be noted. First, this microarray data only assessed 10 samples, and this small sample size reduces the robustness in detecting more significant miRNAs. Second, the clinical features of the IDD in this study are limited, and some other important clinical features, such as injuries and nutrition, were not analyzed, and thus we could not explore their correlation to miRNA expression. Third, this study solely performed a bioinformatics analysis of miRNA microarray data, and the results were not validated using *in vivo* and *in vitro* experiments. Fourth, the effect of miRNAs should be further investigated *via* their target genes; however, due to insufficient research funds, functional studies were not performed. Therefore, further investigations on the function of these targeted genes are warranted.

## CONCLUSION

The present study provides a system biology perspective in characterizing miRNA expression in IDD. We have identified some miRNAs that are both differentially expressed and closely related to the IDD stage. The function of hub miRNAs in the co-expression network analysis is also elucidated, suggesting their potential use as therapeutic targets for IDD. Further experimental studies validating the role of these miRNAs in IDD should be performed.

## Figures and Tables

**Table 1:** Primer sequences used in qRT-PCR analysis.

**-**	**-**	**Primer Sequence**
Hsa-miR-1827	Forward	5'-CGGTGAGGCAGTAGATTGAATAA-3'
-	Reverse	5'-GCG AGC ACA GAATTA ATACGAC-3'
Hsa-miR-185-5p	Forward	5'-AGGGGCUGGCUUUCCUCUGGUC-3'
-	Reverse	5'-GCGAGCACAGAATTAATACGAC-3'
Hsa-miR-4534	Forward	5'-GGGATGGAGGAGGGGTCTAA-3'
-	Reverse	5'-GCGAGCACAGAATTAATACGAC-3'
U6	Forward	5'-GCGCGTCGTGAAGCGTTC-3'
-	Reverse	5'-GTGCAGGGTCCGAGGT-3'

**Table 2:** Characteristics of clinical specimens collected in this study.

**Clinical Specimen**	**IDD**	**Controls**	***P* value**
Stage	15 grade IV, 3 grade V	5 grade II	-
Age	61.5 ± 8.3	56.7 ± 7.4	0.186
Sex	13/5	3/2	0.843
Hsa-miR-1827	0.92 ± 0.17	2.19 ± 0.21	< 0.01
Hsa-miR-185-5p	0.58 ± 0.13	0.82 ± 0.16	< 0.01
Hsa-miR-4534	1.07 ± 0.14	4.45 ± 0.59	< 0.01

## Data Availability

Not applicable.

## LIST OF ABBREVIATIONS

ABCC1: ATP-Binding Cassette Subfamily C Member 1
BCL2: CL2, Apoptosis Regulator
CRKL: CRK-like Proto-Oncogene, an Adaptor Protein
GO: Gene Ontology
IDD: Intervertebral Disc Degeneration
KEGG: Kyoto Encyclopedia of Genes and Genomes
RBX1: Ring-Box 1
WGCNA: Weighted Gene Co-Expression Network Analysis
