# Supplementary material for: Identification and Verification of Key MiRNAs Associated with Intervertebral Disc Degeneration
Source: Comb Chem High Throughput Screen. 2023 Apr 27;26(9):1766–74. doi: 10.2174/1386207325666220915113438 (PMC10242762; doi:10.2174/1386207325666220915113438)
Supplement: Supplementary file 1 [file CCHTS-26-1766_SD1.pdf]

Supplementary Material

Identification and Verification of Key MiRNAs Associated with Intervertebral Disc Degeneration

Jianwei Liu[1,*], Rong Li[2,*] and Peizhen Lyv[1]

[1]*Department of Osteology, The Second Nanning People's Hospital, The Third Affiliated Hospital of Guangxi Medical University, Nanning 530031, Guangxi, China;* [2]*Department of Reproductive Center, The Second Nanning People's Hospital, The Third Affiliated Hospital of Guangxi Medical University, Nanning, 530031, Guangxi, China*

Table S1. Significantly expressed miRNAs that associated with IDD stage.

miRNA_ID	q value.
hsa-miR-4769-5p	2.49E-05
hsa-miR-4534	0.000788971
hsa-miR-1827	0.000889325
hsa-miR-4306	0.002066578
hsa-miR-4656	0.002684023
hsa-miR-185-5p	0.003237166
hsa-miR-4741	0.006007746
hsa-miR-5008-3p	0.007687152
kshv-miR-K12-12-3p	0.008826336
kshv-miR-K12-2-5p	0.01045331
hsa-miR-3648	0.011026095
hcmv-miR-US33-5p	0.011210828
hsa-miR-3945	0.012649938
hsv1-miR-H6-5p	0.015495567
hsa-miR-3196	0.015926392
ebv-miR-BART19-3p	0.016526765
hsa-miR-302c-5p	0.018404299
bkv-miR-B1-5p	0.01884695
hsa-miR-4497	0.019204281
hsa-miR-431-5p	0.019795583
hsa-miR-183-3p	0.020819565
hsa-miR-642b-3p	0.021081373
hsa-miR-4425	0.023606051
hsa-miR-371a-5p	0.024293649
hsa-miR-4533	0.024674926
hsa-miR-328-5p	0.025354549
hsa-miR-5100	0.025394272

hsa-miR-631	0.025395602
hsa-miR-1538	0.026643988
hsa-miR-3158-5p	0.027306758
hsa-let-7f-1-3p	0.030580761
hsa-miR-4747-5p	0.032365
hsa-miR-4748	0.035473201
hsa-miR-204-3p	0.035596193
hsa-miR-4674	0.036774427
hsa-miR-585-3p	0.038452512
hsa-miR-4317	0.038508974
hsa-miR-663a	0.039002488
hsa-miR-208a-5p	0.039987103
hiv1-miR-H1	0.040582768
ebv-miR-BART6-3p	0.041562761
hsa-miR-548at-5p	0.043004604
hsa-miR-4731-3p	0.04303108
hsa-miR-486-5p	0.043056324
hsa-miR-4787-3p	0.045128288
hsa-miR-625-3p	0.047847947
hsa-miR-4419a	0.049788603
hsa-miR-296-5p	0.049990848

Table S2. Differentially expressed miRNAs between normal and degenerated nucleus pulposus tissues.

ID	adj.P.Val	P.Value	logFC	miRNA_ID
46258	0.09329	0.00054438	4.849102	hsa-miR-1184
145838	0.09329	0.00044391	4.6927	hsa-miR-125b-1-3p
148661	0.14303	0.0080018	4.39189	hsa-miR-486-3p
148384	0.09329	0.00061356	3.763601	hsa-miR-3648
145889	0.2053	0.01354683	3.434128	hsa-miR-196b-5p
17520	0.26769	0.0271325	3.223718	hcmv-miR-US5-2-3p
10964	0.22606	0.0175384	3.222864	hsa-miR-155-5p
147796	0.11001	0.00402083	3.01603	hsa-miR-4327
169015	0.22606	0.01780317	2.969762	hsa-miR-4454
17561	0.09329	0.00064835	2.943616	ebv-miR-BART6-3p
168870	0.29498	0.03630563	2.9216	hsa-miR-1246
46443	0.22626	0.01867034	2.65078	hsa-miR-193a-5p
42811	0.10665	0.00298923	2.608877	hsa-miR-542-5p
46326	0.27418	0.03095205	2.594579	hsa-miR-1233-3p

168925	0.10279	0.00194021	2.592945	hsa-miR-1273g-3p
46479	0.13135	0.0068888	2.569756	hsa-miR-1304-5p
168776	0.22299	0.0152815	2.48218	hsa-miR-4795-3p
148032	0.27418	0.03106055	2.443055	hsa-miR-3685
46556	0.26769	0.02733083	2.290602	hsa-miR-623
169328	0.10279	0.00091784	2.284078	hsa-miR-4769-5p
168973	0.27356	0.03022581	2.236804	hsa-miR-1268b
46810	0.10449	0.00237317	2.190244	hsa-miR-1827
17499	0.33029	0.04434682	2.073088	hcmv-miR-US5-1
17810	0.34625	0.04893978	2.021043	hsa-miR-29b-1-5p
168878	0.15648	0.0090823	1.987467	hsa-miR-5100
147203	0.22531	0.01699907	1.925318	hsa-miR-302a-3p
145745	0.3089	0.0403952	1.862892	hsa-miR-335-3p
147722	0.10665	0.00380366	1.81941	hsa-miR-4306
46221	0.29524	0.03654416	1.811335	hsa-miR-519d-3p
11141	0.27391	0.0305028	1.784995	hsa-miR-509-3p
147845	0.22443	0.01561563	1.706725	hsa-miR-3173-3p
32946	0.22531	0.01675904	1.641332	hsa-miR-486-5p
168769	0.27188	0.02929451	1.590811	hsa-miR-5002-5p
42902	0.20801	0.01396457	1.530935	hsa-miR-185-5p
168832	0.24353	0.02257281	1.517568	hsa-miR-4674
46924	0.27037	0.02796582	1.514541	hsa-miR-1252-5p
42522	0.24353	0.02316046	1.454341	ebv-miR-BART19-3p
168639	0.32235	0.04300755	1.154912	hsa-miR-4533
147682	0.34625	0.04932263	-1.117948	hsv1-miR-H6-5p
169322	0.30121	0.03833547	-1.131529	hsa-miR-4534
169388	0.32235	0.04305476	-1.302578	hsa-miR-663a
169059	0.29458	0.03605007	-1.408304	hsa-miR-642a-3p
168642	0.23271	0.01985385	-1.435268	hsa-miR-642b-3p
169221	0.28851	0.03450027	-1.481441	hsa-miR-4748
42700	0.22606	0.01772632	-1.629996	hsa-miR-631
147919	0.13461	0.00724823	-1.659561	kshv-miR-K12-12-3p
168673	0.11001	0.00423958	-1.720715	hsa-miR-4656
42812	0.28753	0.0332152	-1.804458	hsa-miR-508-5p
147817	0.12037	0.00612471	-1.852047	hsa-miR-3196
168631	0.22606	0.01806748	-1.877778	hsa-miR-4723-5p
148351	0.12037	0.00614458	-1.897775	hsa-miR-3945
169355	0.27391	0.03064696	-1.933324	hsa-miR-2355-5p
145905	0.27188	0.02932782	-1.946249	hsa-miR-518a-5p,hsa-miR-527

168898	0.11522	0.00552206	-1.972193	hsa-miR-5008-3p
147739	0.28031	0.03214732	-2.010832	hsa-miR-3161
168753	0.11107	0.00473801	-2.017463	hsa-miR-4741
17953	0.1861	0.01119189	-2.099043	hsa-miR-183-3p
168646	0.22929	0.01924145	-2.134774	hsa-miR-4731-3p
147776	0.10665	0.00360103	-2.222775	hsa-miR-4317
11121	0.22443	0.01587844	-2.260343	hsa-miR-489-3p
169306	0.10665	0.00340164	-2.289313	hsa-miR-4758-5p
169043	0.28753	0.03388508	-2.310085	hsa-miR-4462
145705	0.27252	0.02992032	-2.342685	hsa-miR-431-5p
42501	0.10279	0.00212345	-2.351635	hcmv-miR-US33-5p
42571	0.33438	0.04583086	-2.357496	hsa-miR-129-1-3p
17470	0.12859	0.00665415	-2.390769	kshv-miR-K12-2-5p
46684	0.1358	0.00750217	-2.391547	hsa-miR-1225-3p
169326	0.23108	0.01955252	-2.403087	hsa-miR-451b
168564	0.10279	0.00204276	-2.436109	hsa-miR-548at-5p
168923	0.27037	0.02826845	-2.473114	hsa-miR-4688
148674	0.24353	0.02188126	-2.56822	hsa-miR-4321
148509	0.10665	0.00295452	-2.593749	hsa-miR-328-5p
169245	0.11311	0.00490416	-2.65853	hsa-miR-2467-3p
42477	0.30121	0.0381008	-2.738087	hsa-miR-324-5p
169218	0.29535	0.03676396	-2.79885	hsa-miR-5189-5p
145643	0.14344	0.00812473	-2.830949	hsa-miR-382-5p
168597	0.30237	0.03931551	-2.925841	hsa-miR-5699-3p
148673	0.19334	0.0119721	-2.934704	hsv1-miR-H15
147818	0.30237	0.03932874	-2.935444	hsa-miR-4270
29562	0.20252	0.01317107	-2.98138	hsa-miR-199a-5p
148657	0.24353	0.02296226	-2.99963	hsa-miR-381-5p
146185	0.10279	0.00217882	-3.110239	bkv-miR-B1-5p
46450	0.22531	0.01701629	-3.136218	hsa-miR-548o-3p
169108	0.10279	0.00156069	-3.147315	hsa-miR-4713-5p
148650	0.06542	0.00022873	-3.154402	hsa-miR-516a-5p
168964	0.10279	0.00222831	-3.24153	hsa-miR-4450
147996	0.11001	0.00445209	-3.354886	hsa-miR-3127-5p
148635	0.13549	0.00739046	-3.431393	hsa-miR-933
168684	0.2674	0.02655267	-4.033574	hsa-miR-5010-5p
145724	0.05002	0.00010493	-4.054256	hsa-miR-887-3p
17338	0.10279	0.00199004	-4.803983	hsa-miR-660-5p
46863	0.18358	0.01091208	-4.901346	hsa-miR-3157-3p

147979	0.00885	0.00001238	-5.029311	hsa-miR-3150a-3p
147938	0.11001	0.00449716	-5.808237	hsa-miR-4287
